# High-Level Oxygen Reduction Catalysts Derived from the Compounds of High-Specific-Surface-Area Pine Peel Activated Carbon and Phthalocyanine Cobalt

**DOI:** 10.3390/nano11123429

**Published:** 2021-12-17

**Authors:** Lei Zhao, Ziwei Lan, Wenhao Mo, Junyu Su, Huazhu Liang, Jiayu Yao, Wenhu Yang

**Affiliations:** 1 Department of Physical Science and Technology, Lingnan Normal University, Zhanjiang 524048, China; momlanzw@foxmail.com (Z.L.); mowenhaobiaodi@126.com (W.M.); w1137615926@163.com (J.S.); atiao0606@163.com (H.L.); jiayu_yao2000@163.com (J.Y.); 2 School of Electronics and Information Engineering, Guangdong Ocean University, Zhanjiang 524088, China

**Keywords:** oxygen reduction reaction, nanoporous activated carbon, cobalt-nitrogen-doped carbon, nonprecious metal catalyst

## Abstract

Non-platinum carbon-based catalysts have attracted much more attention in recent years because of their low cost and outstanding performance, and are regarded as one of the most promising alternatives to precious metal catalysts. Activated carbon (AC), which has a large specific surface area (SSA), can be used as a carrier or carbon source at the same time. In this work, stable pine peel bio-based materials were used to prepare large-surface-area activated carbon and then compound with cobalt phthalocyanine (CoPc) to obtain a high-performance cobalt/nitrogen/carbon (Co-N-C) catalyst. High catalytic activity is related to increasing the number of Co particles on the large-specific-area activated carbon, which are related with the immersing effect of CoPc into the AC and the rational decomposed temperature of the CoPc ring. The synergy with N promoting the exposure of CoN_x_ active sites is also important. The E_onset_ of the catalyst treated with a composite proportion of AC and CoPc of 1 to 2 at 800 °C (AC@CoPc-800-1-2) is 1.006 V, higher than the Pt/C (20 wt%) catalyst. Apart from this, compared with other AC/CoPc series catalysts and Pt/C (20 wt%) catalyst, the stability of AC/CoPc-800-1-2 is 87.8% in 0.1 M KOH after 20,000 s testing. Considering the performance and price of the catalyst in a practical application, these composite catalysts combining biomass carbon materials with phthalocyanine series could be widely used in the area of catalysts and energy storage.

## 1. Introduction

Environmental pollution and foreseeable energy shortages have become a tricky problem, so pollution-free and renewable energy technology is of paramount importance to mankind today [1,2,3]. The oxygen reduction reaction (ORR) is a critical central reaction in fuel cells and metal-air batteries, but fuel cells are always limited for the sluggishness of the ORR at the cathode [4,5,6]. Nowadays, the predominant commercial ORR catalysts are still made of platinum (Pt) and its alloys because of their outstanding properties. However, Pt and its alloys are expensive and scarce in resources, which greatly limits their practical applications [7,8,9].

In recent years, in order to overcome the shortcomings of Pt-based catalysts, many nonprecious metal ORR catalysts with high electrocatalytic activity have been explored, including metal hydroxides [10], oxides [11], sulfides [12], phosphides [13], nitrides [14], selenides [15], and heteroatom-doped carbon materials [16,17,18]. Among them, heteroatom-doped carbon materials are very effective in improving the catalytic activity of the ORR with a high SSA and lots of catalytic sites [19]. Doping carbon with heteroatoms (especially N) can regulate the electron distribution of carbon atoms, as a result of higher catalytic activity and stability [20]. It is also reported that the pyridine-like and pyrrole-like N is at the origin of the excellent ORR catalytic activity [21,22]. Besides, transition metals (for example, Co, Fe, and Ni) also have a pivotal role in the ORR. For instance, M-N-C composite catalysts composed of N-doped carbon materials and transition metal have been widely studied for their abundant active sites and high conductivity [23,24,25]. Cobalt phthalocyanine (CoPc) derivatives are effective catalysts for the ORR [26]. However, inactive dimers of the phthalocyanine complexes formed in solution will significantly affect their catalytic properties. An efficient way is to develop hybrid catalysts based on carbon [27,28]. Compared with many carbon materials, biomass-derived carbon materials have been developed as low-cost nonprecious metal catalysts thanks to their high availability, accessibility, and recyclability. Biomass pine peels are widely distributed and easily available, which will provide a good foundation for the development and application of nonprecious metal N-doped carbon catalysts. Then, designing porous nanostructured carbon materials with a high SSA using pine peels is critical to obtain high-performance catalysts [29,30].

In this work, we synthesized a series of AC/CoPc composite catalysts through pre-activation treatment, high-temperature carbonization methods, and precise control of the composite proportion of AC derived from biomass pine peel and CoPc. The AC/CoPc-800-1-2 catalyst carbonized at 800 ℃, in which AC and CoPc were mixed with the mass ratio of 1:2, showed excellent catalytic ability for the ORR. Compared to other precursors containing nitrogen and cobalt (such as aniline [31], melamine [32], pyrrole [33], and metal organic frameworks [34]), this method is simpler, more effective, lower cost, and more promising to prepare Co-N-C catalysts with a synergistic effect and significantly enhanced ORR catalytic activity.

## 2. Experimental

### 2.1. Materials

All chemical reagents were of analytical grade and used without further purification. The pine peel came from the forest area of Little Xing’an Mountain in Heilongjiang Province, China. The tree species is Pinus Koraiensis Siebold et Zuccarini. Its growing soil is black soil, with slow growth and stable structure, making nitrogen more stable in the inside of the tree. Potassium hydroxide (KOH), hydrochloric acid (HCl), cobalt phthalocyanine (CoPc), and ethanol were purchased from Shanghai Macklin Biochemical Co., Ltd. (Shanghai, China). High-purity N_2_ and O_2_ were supplied by the Zhanjiang Zhantong Industrial Gases Co., Ltd. (Zhanjiang, China). Deionized water was used for all experiments.

### 2.2. Preparation of AC/CoPc Series Composite Catalysts

#### 2.2.1. Preparation of AC

Firstly, the pine peel was washed with deionized water and crushed. Then, we mixed the pine peel with KOH at a mass ratio of 1:4 and heated it at 900 ℃ for 1 h in a tubular furnace (OTF-1200X, Hefei Kejing Co., Ltd. (Hefei, China)) with the heating rate of 5 ℃ min^−1^ under a N_2_ atmosphere with a flowing rate of 100 mL min^−1^. After activation, the above samples were washed with 1 M HCl and distilled water several times and dried for 12 h at 60 °C. Finally, the obtained samples were denominated as AC. 

#### 2.2.2. Preparation of AC/CoPc

**Scheme 1:**
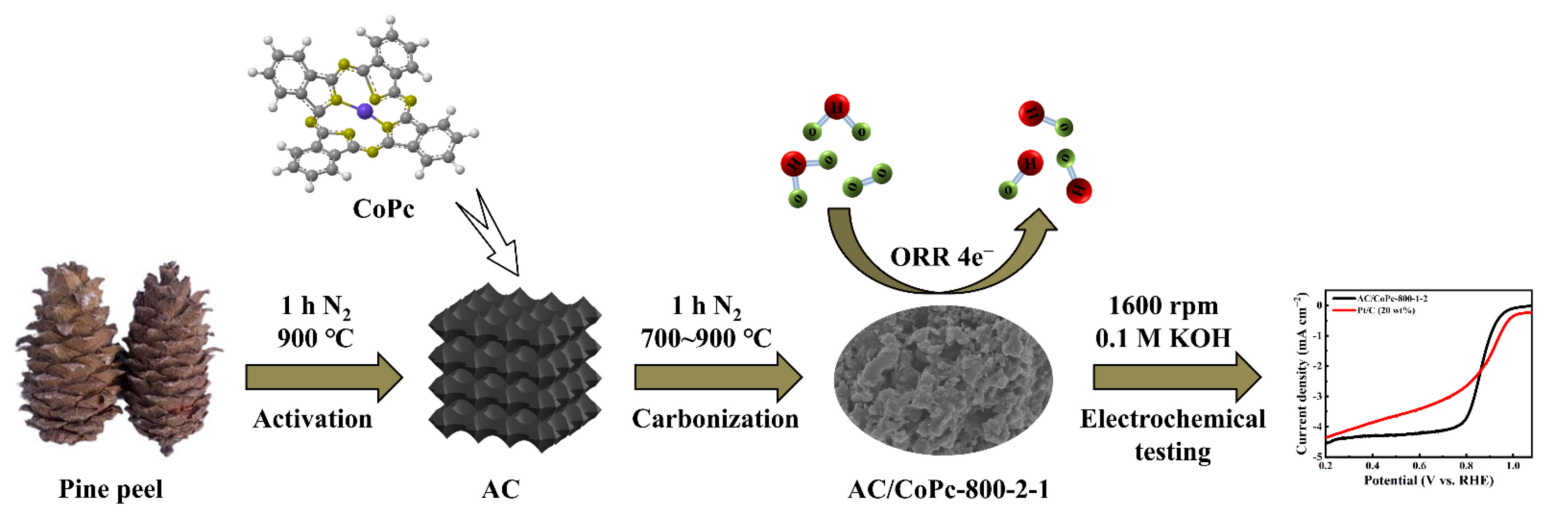
The schematic illustration of the synthesis of AC/CoPc series catalysts.

To obtain AC/CoPc series composite catalysts, AC and CoPc were mixed with the mass ratios of 2:1, 1:1, and 1:2 by carbonizing at 700, 800, and 900 ℃ for 1 h in a N_2_ atmosphere, respectively. Then the obtained samples were ground for 1 h with ethanol solution in a glass dish, and dried for 12 h at 60 °C. Scheme 1 is a schematic illustration of AC/CoPc series composite catalysts. The AC/CoPc series composite catalysts were named as a self-defined pattern; for example, the composed materials of AC and CoPc mixed with the mass ratio of 2:1 by carbonizing at 700 ℃ were denominated as AC/CoPc-700-2-1.

### 2.3. Structure Characterizations

Scanning electron microscopy (SEM, Hitachi S-4800, Hitachi, Tokyo, Japan) was used to investigate the surface morphology and structure of the catalyst samples. X-ray diffraction (XRD, XRD-6000, Shimadzu, Kyoto, Japan) patterns of the samples were obtained on an XRD-6000 X-ray diffractometer using Cu K_a_ radiation with 4° min^−1^. Transmission electron microscopy (TEM, JEM-2100F, JEOL, Tokyo, Japan) and selected area mapping were collected and were operated on a JEM-2100F instrument with an acceleration voltage of 100 kV. X-ray photoelectron spectroscopy (XPS, ESCALAB 250 iXL, Thermo Fisher Scientific, Massachusetts, America) analysis was performed using an ESCALAB 250 iXL spectrometer with an Al K_a_ X-ray source. 

### 2.4. Electrochemical Characterizations

Electrochemical experiments were conducted at room temperature on an electrochemical workstation (RST5200F, Zhengzhou Shiruisi Instrument Co., Ltd., Zhengzhou, China). Linear sweep voltammetry (LSV) and rotating-disk electrode (RDE) polarization curves were measured in a conventional three-electrode electrochemical system. A platinum wire (CHI115) electrode and Ag/AgCl (sat.) (CHI111) electrode were used as counter electrode and reference electrode, respectively. A glassy carbon (GC) electrode (5 mm in diameter, 0.196 cm^2^) was used for the working electrode to test the LSV and RDE curves. Before measurements, the GC electrodes were carefully polished with gamma alumina powders (0.05 mm) until a mirror-like surface was obtained, and then washed with distilled water twice and dried in a vacuum. Subsequently, the AC/CoPc series composite catalysts (400 μg cm^−2^) were put onto the GC electrode followed by dripping a drop of Nafion solution (5 wt%, Dupont), improving the adhesion of active materials and the GC electrode surface. All electrode potentials in this work were quoted versus a reversible hydrogen electrode (vs. RHE), and a potential of 0.989 V was added to the conversion with RHE. RDE experiments for ORR were performed over the potential range of 0.2~1.1 V in O_2_-saturated 0.1 M KOH solution at the scan rate of 10 mV s^−1^. The RDE polarization curves were measured by the reference electrode of Ag/AgCl (sat.) in 0.1 M KOH solution at the scan rate of 10 mV s^−^^1^ and rotation rates from 400 to 1600 rpm. Measurements of the current–time (i–t) curves were used to evaluate the stability of the catalyst at a constant potential of 0.6 V (vs. RHE) for 20,000 s, in which O_2_ was bubbled at a continuous flow rate of 20 mL min^−1^ at the rotation rate of 1600 rpm. Under the same experimental conditions, Pt/C (20 wt%) purchased from Shanghai He Sen Electric Co., Ltd. (Shanghai, China) was used for the above experimental comparison.

### 2.5. Calculation of Electron Transfer Number (n)

The electron transfer number (*n*) is determined by the Koutecky–Levich equation at a series of potentials:

$$\frac{1}{J}=\frac{1}{J_{L}}+\frac{1}{J_{K}}=\frac{1}{B\omega^{1/2}}+\frac{1}{J_{K}}$$

*B* = *0.62FC*_0_(*D*_0_)^2/3^*ν*^−1/6^

*J*_*K*_ = *nFkC*_0_

where *J* is the measured current density (mA cm^−2^), *J_L_* and *J_K_* are the diffusion-limiting and kinetic current densities (mA cm^−2^), *ω* is the angular velocity of the disk (*ω = 2πN*, *N* is the linear rotation speed), *n* is the overall number of electrons transferred per oxygen molecule during ORR, *D*_0_ is the diffusion coefficient (cm s^−1^), *F* is the Faraday constant (F = 96,486.4 C mol^−1^), *C*_0_ is the bulk concentration of O_2_ (mol L^−1^), *v* is the kinematic viscosity of the electrolyte, *k* is the electron transfer rate constant, and the values of *C*_0_, *D*_0_, and *v* for O_2_-saturated 0.1 M KOH solution are 1.20 × 10^−6^ mol cm^−3^, 1.90 × 10^−5^ cm^2^ s^−1^, and 0.01 cm^2^ s^−1^, respectively.

## 3. Results and Discussion

**Figure 1:**
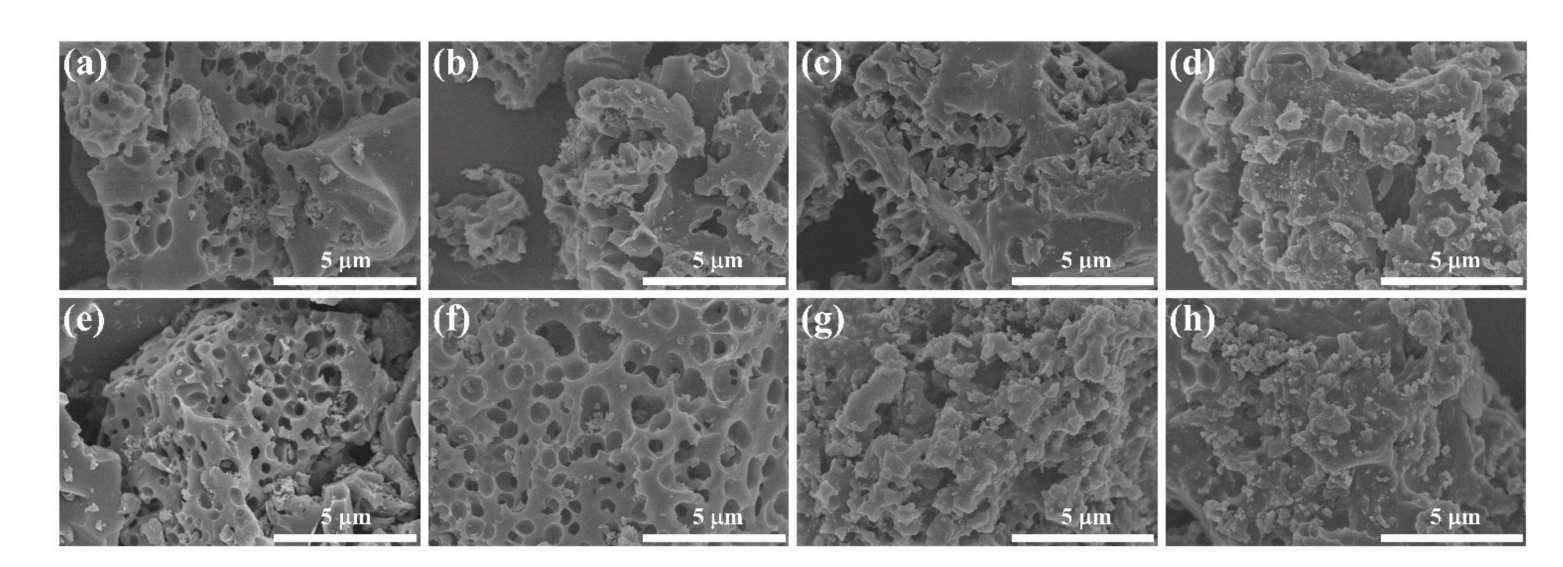
SEM images with low magnification: (**a**) AC, (**b**) AC/CoPc-700-2-1, (**c**) AC/CoPc-700-1-1, (**d**) AC/CoPc-700-1-2, (**e**) AC/CoPc-800-2-1, (**f**) AC/CoPc-800-1-1, (**g**) AC/CoPc-800-1-2, and (**h**) AC/CoPc-900-1-2.

**Figure 2:**
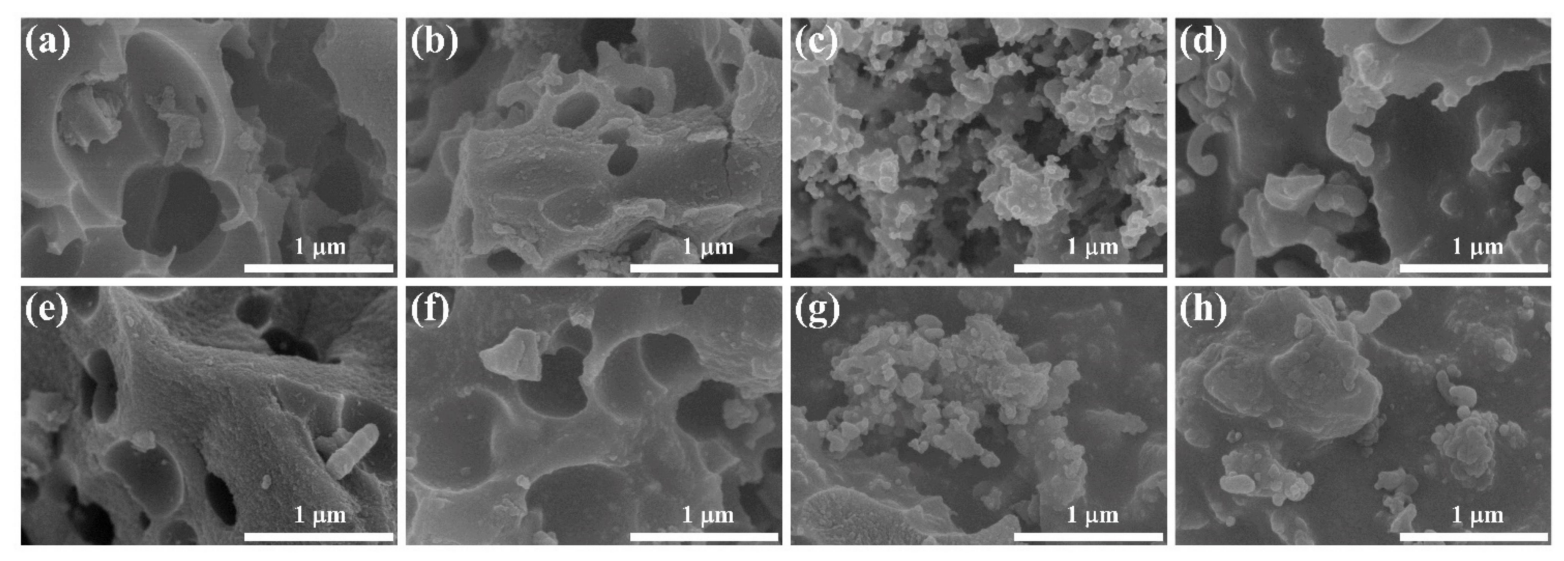
SEM images with high magnification: (**a**) AC, (**b**) AC/CoPc-700-2-1, (**c**) AC/CoPc-700-1-1, (**d**) AC/CoPc-700-1-2, (**e**) AC/CoPc-800-2-1, (**f**) AC/CoPc-800-1-1, (**g**) AC/CoPc-800-1-2, and (**h**) AC/CoPc-900-1-2.

The SEM images of AC and different proportion AC/CoPc series composite catalysts are shown in Figure 1 and Figure 2. In Figure 1b,e, it should be due to the low proportion of composite CoPc, which generally shows a basic nanoporous structure, as in AC (Figure 1a). As the temperature and the proportion of composite CoPc increase, the surface nanoporous structure gradually decreases, and the particles attached to the surface gradually increase (Figure 1b–h), where AC/CoPc-800-1-2 (Figure 1g) shows a uniform distribution. As can be seen from Figure 2, with the increase in temperature, CoPc compound can prevent the formation of a nanoporous structure, and the SSA will also change during the heating process of 700~900 ℃ (Figure 2b–h). Compared with AC/CoPc-900-1-2 (Figure 2h), AC/CoPc-800-1-2 (Figure 2e) has a better composite degree of AC and CoPc, with more uniform distribution and higher SSA. These results suggest that the rich distribution and size of nanopores can be regulated and controlled by pyrolysis temperature and activation, thereby forming more nanopores to expose more active areas and promote the ability of electron transfer [35,36], but the appropriate temperature and proportion are more conducive to the recombination of AC and CoPc, as well as providing more adhesion sites for CoPc.

**Figure 3:**
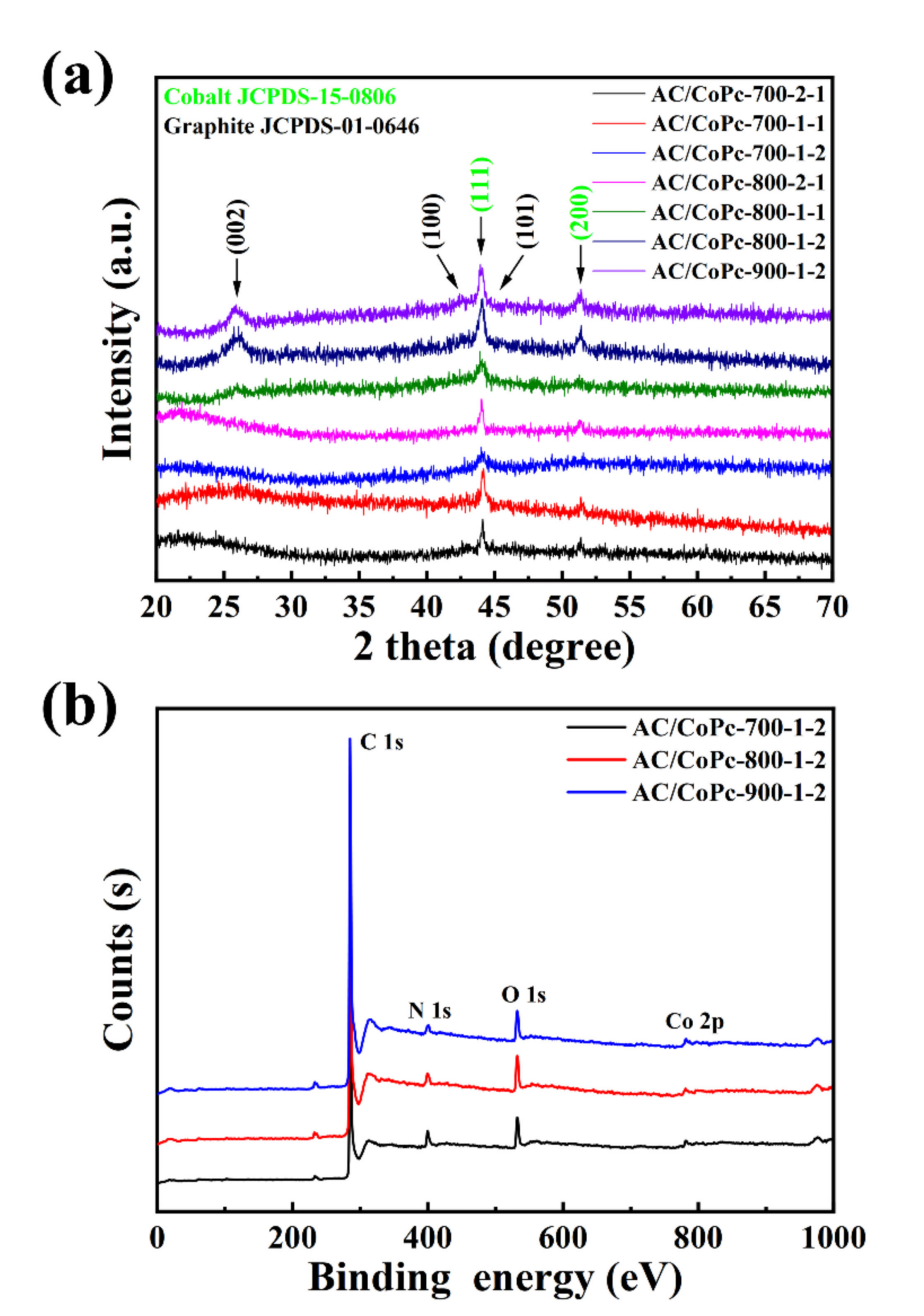
(**a**) The XRD patterns of AC/CoPc series catalysts, (**b**) the XPS survey spectra of AC/CoPc-700-1-2, AC/CoPc-800-1-2, and AC/CoPc-900-1-2.

The XRD patterns of AC/CoPc series composite catalysts (Figure 3a) all correspond to Co (JCPDS 15-0806) [37] and graphite (JCPDS 01-0646) [38]. AC/CoPc-800-1-2 and AC/CoPc-900-1-2 exhibit that the broadened peak at 25.7° is ascribed to the (002) plane of graphite (JCPDS 01-0646), but AC/CoPc-800-1-2 has a larger broad peak at 25.7°, which manifests that the carbonization temperature of 800 ℃ is more conducive to the formation of carbon with small graphite domains [39]. Moreover, the peaks of AC/CoPc series composite catalysts at 44.2° and 51.5° can be indexed to the (111) and (200) plane of Co (JCPDS 15-0806), indicating that CoPc has been transformed into metallic cubic-phase Co nanoparticles under high-temperature pyrolysis, which can be reflected from the following XPS analysis.

**Figure 4:**
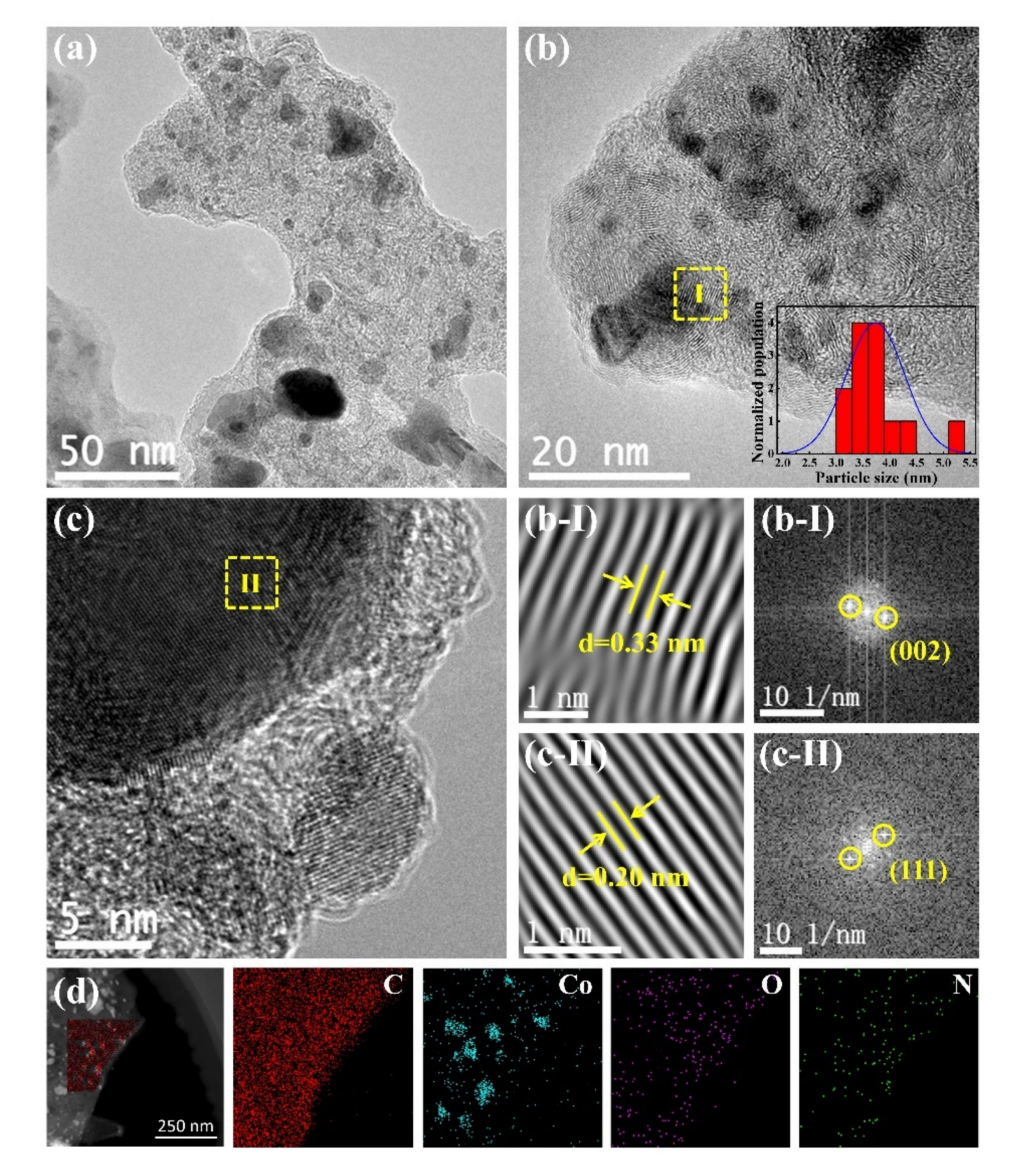
(**a**,**b**) The TEM and (**c**) HRTEM images of AC/CoPc-800-1-2, the FFT filtered TEM images of different selected regions (I and II), (**d**) the corresponding elemental mapping analysis of AC/CoPc-800-1-2, and the illustration in the lower right corner of Figure 4b is the size distribution of Co nanoparticles.

TEM was used to further characterize the structural details of AC/CoPc-800-1-2. In Figure 4a, many vague small black spots are exposed as active sites on the carbon skeleton, although very few of them are clustered together; most of them are uniformly distributed. It can be seen more clearly from Figure 4b that these small black spots are Co nanoparticles with a particle size concentrated at about 3.5~4.0 nm. The FFT filtered TEM images of Figure 4b and c confirm that these active sites are Co nanoparticle active sites with a lattice spacing of 0.20 nm, corresponding to the (111) crystal plane of Co, which have been embedded into the carbon skeleton, while 0.33 nm corresponds to the (002) crystal plane of graphite [40]. This value is larger than the spacing of (002) in graphite, showing a disordered effect in the catalyst, and the graphite carbon tightly wraps the active sites of Co nanoparticles in the carbon skeleton, which also enhances the mechanical stability of nanostructured composites [41,42]. The element mappings (Figure 4d) display the good dispersion of C, O, N, and Co, which is the result of the N-doped carbon with interspersed Co.

**Figure 5:**
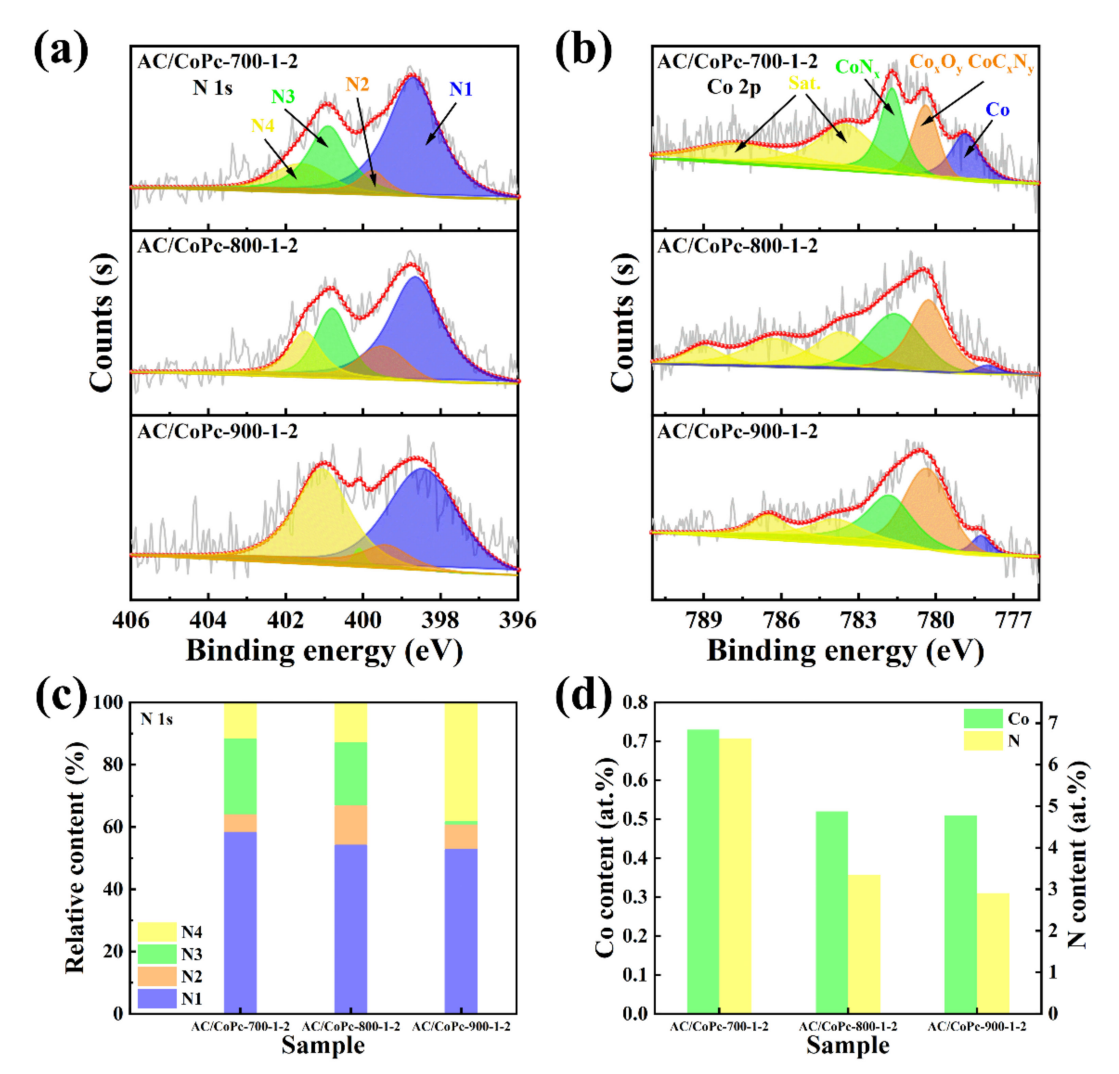
The XPS spectra of (**a**) N 1s, (**b**) Co 2p, (**c**) the relative contents of different N species obtained from fitting of XPS spectra of N 1s, and (**d**) surface content of N and Co obtained from XPS survey spectra for AC/CoPc-700-1-2, AC/CoPc-800-1-2, and AC/CoPc-900-1-2.

The XPS spectra of AC/CoPc-700-1-2, AC/CoPc-800-1-2 and AC/CoPc-900-1-2 are given in Figure 3b, including four elements C, O, N, and Co. The major part of pyridinic-N (N1) moieties in AC/CoPc-700-1-2 (Figure 5a) is 54.87% but two peaks at 399.7 eV (N2, CoN_x_) and 400.9 eV (N3, pyrrolic-N) are relatively lower compared with AC/CoPc-800-1-2 (Figure 5c). Another two peaks at 401.3 eV and reported in the literature from 397 to 399.5 eV are assigned to graphitic-N (N4) and pyridinic-N (N1) [43]. The major part of nitrogen moieties in AC/CoPc-800-1-2 exhibits the higher contribution of pyridine-N and a high amount of Co and N association in the CoN_x_ structure. Except the two catalysts mentioned above, AC/CoPc-900-1-2 shows lower CoN_x_ and pyrrolic-N content in Table 1. Therefore, we have reason to infer that pyridine-N sites and CoN_x_ have a substantial role in ORR. For Co 2p, the XPS spectra of these three composite catalysts (Figure 5b) show that three main peaks at 780.3 eV, 781.8 eV, and around 783 eV are assigned to Co, Co_x_O_y_ or CoC_x_N_y_, and CoN_x_ respectively [44,45,46]. The cobalt content percentages of these three composite catalysts are 0.77%, 0.52%, and 0.51%, respectively. As the temperature increased, the cobalt content gradually decreased, while the nitrogen content was 6.63%, 3.35%, and 2.91%, also showing a downward trend (Figure 5d). This indicates that high temperature (800 °C) can increase the reaction rate between Co and N, but too high a temperature (900 °C) will cause a large loss of N. Compared with AC/CoPc-700-1-2 and AC/CoPc-900-1-2, cobalt content in AC/CoPc-800-1-2 shows higher CoN_x_ content and the result is consistent with the analysis of the N2 moiety.

**Figure 6:**
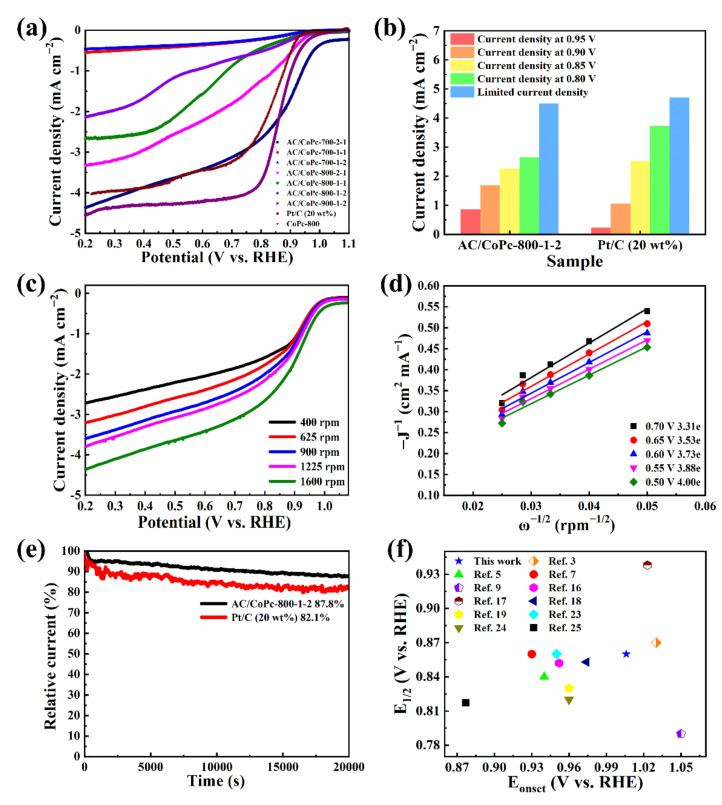
(**a**) RDE curves of AC/CoPc series catalysts, CoPc and Pt/C (20 wt%) catalyst at the rotation speed of 1600 rpm with the scan rate of 10 mV s^−1^ in O_2_ saturated 0.1 M KOH, (**b**) the current density at 0.80~0.95 V (vs. RHE) and limited current densities at 0.30 V (vs. RHE) of AC/CoPc-800-1-2 and Pt/C (20 wt%) catalyst, (**c**) LSV curves of AC/CoPc-800-1-2 at different rotation speeds from 400 rpm to 1600 rpm with the scan rate of 10 mV s^−1^ in O_2_ saturated 0.1 M KOH, (**d**) the corresponding K-L plots (−J^−1^ vs. ω^−1/2^) at different potential in O_2_ saturated 0.1 M KOH, (**e**) stable testing results of AC/CoPc-800-2-1 and Pt/C (20 wt%) catalyst at 0.60 V (vs. RHE) and (**f**) the comparison of the onset potential (V vs. RHE) and half-wave potential (V vs. RHE) in 0.1 M KOH of AC/CoPc-800-1-2 with other Co-N-C catalysts reported in the last three years.

In order to identify the properties of catalysts in Figure 6a, Table 2 contrasts the parameters of AC/CoPc-800-1-2 (the best ORR catalyst in the AC/CoPc series composite catalysts) and Pt/C (20 wt%) catalyst. The difference between the ORR activities of AC/CoPc series composite catalysts was compared, including E_onset_ and E_1/2_, as well as the current densities of 0.95, 0.90, 0.85 and 0.80 V. As shown in Figure 6a, the E_onset_ of AC/CoPc-800-1-2 is 1.006 V, which is higher than Pt/C (20 wt%) catalyst with the onset potential of 0.989 V. The pyrolyzed CoPc alone at 800 °C does not show a high current density and half-wave potential in comparison with the AC/CoPc-800-1-2 catalyst in Figure 6a. These results demonstrate that except for the limited current density (Figure 6b), the AC/CoPc series composite catalysts show better catalyst effects than Pt/C (20 wt%) catalysts. The LSV curves at different rotating speeds were tested to further evaluate the ORR performance of AC/CoPc-800-1-2 (Figure 6c), and the corresponding K-L plots are given in Figure 6d. The K-L plots show good linearity and parallelism, indicating that the ORR process of the AC/CoPc-800-1-2 follows first-order kinetics in the selected potential range from 0.50 to 0.70 V (vs. RHE). The electron transfer numbers (n) transferred during ORR and the kinetic limited current density (*J*_k_) can be calculated from the following K-L equation [47,48]. Transfer electron numbers (n) of AC/CoPc-800-1-2 from 0.50 to 0.70 V in Figure 6d are all around four electrons, showing a high four-electron pathway. The stable testing results of AC/CoPc-800-1-2 and commercial Pt/C (20 wt%) catalyst were evaluated by i–t curve at a constant potential of 0.6 V (vs. RHE) with a disk-rotating rate of 1600 rpm (Figure 6e). After 20,000 s testing, the stability of AC/CoPc-800-1-2 was 87.8%, which is much higher than commercial Pt/C (20 wt%) catalyst with only 82.1% stability. Furthermore, the E_onset_ (vs. RHE) and E_1/2_ (vs. RHE) of AC/CoPc-800-1-2 are comparable to those of various Co-N-C catalysts in 0.1 M KOH (Figure 6f), as listed in Table 3.

## 4. Conclusions

In summary, AC/CoPc series composite catalysts with a nanoporous structure were prepared by pre-activation treatment, high-temperature pyrolysis and precise control of the composite proportion of AC and CoPc, in which the AC derived from biomass pine peel served as a carbon carrier compound, with the nitrogen (N) source and inexpensive CoPc serving as the Co and N source. Compared with other AC/CoPc series composite catalysts and Pt/C (20%) catalyst, AC/CoPc-800-1-2 exhibits that the E_onset_ is 1.006 V and the stability is 87.8% in 0.1 M KOH. The high electrocatalytic activity of AC/CoPc-800-1-2 composite catalyst can be attributed to the following three points. (i) AC derived from biomass pine peel is a kind of biomass carbon material with a rich nanoporous structure and high SSA. When used as a carbon carrier, AC can not only provide more attachment sites for CoPc particles, but can also enhance the mechanical stability of the nanostructured composite material and prevent the agglomeration of composite catalyst particles. (ii) Heteroatom N-doped AC ameliorates the charge distribution of adjacent C atoms and optimizes the adsorption of key ORR intermediates, which greatly promotes O_2_ adsorption and electron transfer. (iii) A reasonable composite proportion of AC and CoPc exposes more active sites, so that plentiful atomically dispersed Co nanoparticles encapsulated by graphitic carbon can be formed and synergistically with N promote the exposure of CoN_x_ active sites. More importantly, taking the performance and price of the catalyst in practical application into account, this composite catalyst that directly obtains carbon materials from biomass and combines with phthalocyanine series compounds is likely to be widely used in nonprecious metal catalysts.

## Figures and Tables

**Table 1:** Ration analysis of the peaks in XPS spectra in AC/CoPc series catalysts.

	Element contents (at%)							
Catalysts	C	O	Co	N total	N1	N2	N3	N4
AC/CoPc-700-1-2	85.19	7.45	0.73	6.63	58.47	5.64	24.31	11.58
AC/CoPc-800-1-2	90.04	6.09	0.52	3.35	54.29	12.61	20.33	12.76
AC/CoPc-900-1-2	92.02	4.56	0.51	2.91	53.04	7.79	1.07	38.10

**Table 2:** Comparison of ORR parameters between AC/CoPc-800-1-2 and Pt/C (20 wt%) catalyst.

Catalyst	E_onset_ (V vs. RHE)	E_1/2_ (V vs. RHE)	Current Density at 0.95V (mA cm^−2^)	Current Density at 0.90V (mA cm^−2^)	Current Density at 0.85V (mA cm^−2^)	Current Density at 0.80V (mA cm^−2^)	Limited Current Density (mA cm^−2^)
AC/CoPc-800-1-2	1.006	0.860	0.869	1.688	2.264	2.658	4.50
Pt/C (20 wt%)	0.989	0.858	0.2322	1.060	2.524	3.722	4.70

**Table 3:** Comparison of the content and source of N and Co and ORR catalytic activity of Co-N-C catalysts with values from the literature from the last three years.

Catalysts ^1^	The Content and Source of Co and N (at.%)^2^	E_onset_ (V vs. RHE)	E_1/2_ (V vs. RHE)	Limited Current Density (mA cm^−2^)	Average Transferred Electron Number (*n*)	Durability	Ref.
AC/CoPc-800-1-2	0.52, 3.35 CoPc pine peel	1.006	0.860	4.50	3.69	20,000 s/87.8%	This work
Co-N-C-800	0.83, 2.94 Co(NO_3_)_2_·6H_2_O 2-methylimidazole	1.030	0.870	5.52	3.97	72,000 s/92%	[3]
Co-NC-700	1.07, 4.46 Co(Ac)_2_·4H_2_O 1,10-phenanthroline	0.940	0.840	6.30	3.89	ΔE_1/2_ = −5 mV (10,000 cycles)	[5]
ZIF/ppy-pani-750	1.23, 11.21 Co(NO_3_)_2_·6H_2_O ppy-pani^3^	0.930	0.860	4.99	3.82	64,800 s/88.73%	[7]
ECo@D	0.041, 5.23 EDTA-Co^4^ DA^5^	1.050	0.790	4.74	3.90	36,000 s/93.8%	[9]
Co/N-C@CNFs	4.3, 3.2 Co(NO_3_)_2_·6H_2_O 2-methylimidazole	0.952	0.852	NR	4.20	70,000 s/92%	[16]
Co@N-C-1	NR^6^, 12.12 Co(OAc)_2_urea	1.023	0.938	4.12	3.98	1000 s/80%	[17]
Co-N-CNTs	0.59, 11.93 Co(NO_3_)_2_·6H_2_O 2-methylimidazole	0.974	0.853	NR	4.00	ΔE_1/2_ = 0 mV (10,000 cycles)	[18]
Co@NC-ZM-900	0.61, 1.94 CoPc melamine	0.960	0.830	NR	4.18	20,000 s/94.8%	[19]
Co-NOPC-600	8.74, 4.67 Co(NO_3_)_2_·6H_2_O 2-methylimidazole	0.950	0.860	5.20	3.93	86,400 s/85%	[23]
Co@NC/RGO-2.6	NR, NR Co(NO_3_)_2_·6H_2_O 2-methylimidazole	0.960	0.820	5.60	3.90	ΔE_1/2_ = −2 mV (5000 cycles)	[24]
Co/N-C	0.68, 5.79 Co(NO_3_)_2_·6H_2_O 2-methylimidazole	0.877	0.817	5.11	3.65	36,000 s/87.1%	[25]

^1^ All catalysts measured in 0.1 M KOH solution; ^2^ Co at.% and N at.% were tested by XPS; ^3^ ppy-pani = polypyrrole-polyaniline; ^4^ EDTA-Co = ethylenediaminetetraacetic acid disodium cobalt salt hydrate; ^5^ DA = dopamine hydrochloride. ^6^ NR = Not reported.

## Data Availability

Data are available upon request from the corresponding author.
